# SPIN90 Modulates Long-Term Depression and Behavioral Flexibility in the Hippocampus

**DOI:** 10.3389/fnmol.2017.00295

**Published:** 2017-09-20

**Authors:** Dae Hwan Kim, Minkyung Kang, Chong-Hyun Kim, Yun Hyun Huh, In Ha Cho, Hyun-Hee Ryu, Kyung Hwun Chung, Chul-Seung Park, Sangmyung Rhee, Yong-Seok Lee, Woo Keun Song

**Affiliations:** ^1^Bio Imaging and Cell Logistics Research Center, School of Life Sciences, Gwangju Institute of Science and Technology Gwangju, South Korea; ^2^Department of Physiology, Department of Biomedical Sciences, Seoul National University College of Medicine Seoul, South Korea; ^3^Center for Neuroscience, Korea Institute of Science and Technology, Division of Bio-Medical Science and Technology, KIST School, Korea University of Science and Technology Seoul, South Korea; ^4^Department of Biological Sciences, Dartmouth College Hanover, NH, United States; ^5^Department of Life Science, Chung-Ang University Seoul, South Korea; ^6^Electron Microscope Facility, Dental Research Institute, Seoul National University Seoul, South Korea; ^7^School of Life Sciences, Gwangju Institute of Science and Technology Gwangju, South Korea

**Keywords:** SPIN90 (SH3 protein interacting with Nck, 90 kDa), long-term depression (LTD), behavioral flexibility, synaptic plasticity, learning and memory

## Abstract

The importance of actin-binding proteins (ABPs) in the regulation of synapse morphology and plasticity has been well established. SH3 protein interacting with Nck, 90 kDa (SPIN90), an Nck-interacting protein highly expressed in synapses, is essential for actin remodeling and dendritic spine morphology. Synaptic targeting of SPIN90 to spine heads or dendritic shafts depends on its phosphorylation state, leading to blockage of cofilin-mediated actin depolymerization and spine shrinkage. However, the physiological role of SPIN90 in long-term plasticity, learning and memory are largely unknown. In this study, we demonstrate that *Spin90*-knockout (KO) mice exhibit substantial deficits in synaptic plasticity and behavioral flexibility. We found that loss of SPIN90 disrupted dendritic spine density in CA1 neurons of the hippocampus and significantly impaired long-term depression (LTD), leaving basal synaptic transmission and long-term potentiation (LTP) intact. These impairments were due in part to deficits in AMPA receptor endocytosis and its pre-requisites, GluA1 dephosphorylation and postsynaptic density (PSD) 95 phosphorylation, but also by an intrinsic activation of Akt-GSK3β signaling as a result of *Spin90*-KO. In accordance with these defects, mice lacking SPIN90 were found to carry significant deficits in object-recognition and behavioral flexibility, while learning ability was largely unaffected. Collectively, these findings demonstrate a novel modulatory role for SPIN90 in hippocampal LTD and behavioral flexibility.

## Introduction

Synaptic plasticity manifests persistent changes in synaptic communication, which allows translation of brief experiences into long-lasting memories in the brain (Citri and Malenka, 2008). Proper memory storage is achieved at synapses through long-term potentiation (LTP) and long-term depression (LTD), bidirectional models of plasticity that strengthen or weaken pre-existing synapses (Collingridge et al., 2004; Woolfrey and Dell’Acqua, 2015). During LTP and LTD, microscale protrusions along neuronal dendrites called dendritic spines undergo dynamic enlargement or shrinkage, hence formation or elimination of synapses, based on stimulation received from presynaptic terminals (Bourne and Harris, 2008; Holtmaat and Svoboda, 2009; Lai and Ip, 2013). The symmetry in activity-dependent remodeling of synaptic structure and responses is thought to be the cellular mechanism underlying learning and memory (Holtmaat and Svoboda, 2009).

Actin cytoskeleton is the major mechanical framework at spines, the dynamics of which control various neuronal processes such as spine morphology, neurite outgrowth, trafficking of intracellular components and redistribution of postsynaptic density (PSD) constituents (Okamoto et al., 2004; Winder and Ayscough, 2005; Cingolani and Goda, 2008). Much evidence highlight the critical roles of actin-binding proteins (ABPs) within spines involved in actin assembly or disassembly, allowing for proper sculpting of synaptic structure and plasticity (Yuste and Bonhoeffer, 2001; Winder and Ayscough, 2005; Bourne and Harris, 2008). For example, members of the ADF/cofilin (actin depolymerizing factor/cofilin) family are involved in regulating spine volume and are required for maintenance of LTP and spine shrinkage associated with LTD (Hotulainen et al., 2009; Rust, 2015). The absence of IRSp53 (Insulin receptor substrate protein, 53 kDa), a scaffolding protein implicated in the regulation of actin, leads to enhanced LTP and impaired hippocampus-dependent learning and memory (Kim M. H. et al., 2009). Defects in synaptic plasticity associated with aberrant ABPs have been implicated in several neurological disorders, ranging from Alzheimer’s disease to Autism spectrum disorders (ASDs; Penzes et al., 2011). It is therefore not entirely surprising that the complex interplay between synaptic ABPs and actin dynamics are crucial to mechanisms underlying synaptic plasticity and higher-order brain function.

Although LTP has been established as the cellular correlate of learning, much less have been highlighted about LTD (Malenka and Bear, 2004). Generally, LTD is required to omit synapses from earlier memory traces for new memory to be stored (Nicholls et al., 2008; Mills et al., 2014). LTD in the hippocampus has been implicated in multiple forms of reversal learning, behavioral flexibility, episodic-like memory, immediate memory of a novel context and novelty-detection (Kemp and Manahan-Vaughan, 2004; Nicholls et al., 2008; Kim et al., 2011). Interestingly, this process is thought to be mediated by actin regulated co-factors, which maintain synaptic structure and stability during LTD (Collingridge et al., 2010). A recent report uncovered the roles of actin in fear memory formation and extinction, reflecting the correlation between actin, plasticity and behavioral modification (Lamprecht, 2011). Therefore, clarifying the mechanism by which ABPs modulate structural and functional plasticity in synapses may provide new insight into the role of actin-regulatory proteins in behavioral modification.

SH3 protein interacting with Nck 90 kDa (SPIN90, also known as *NCKIPSD*) is highly expressed in the hippocampus (Kim et al., 2005). Previous studies have shown that SPIN90 acts on actin cytoskeleton, to play a key role during spine formation and the maintenance of spine morphology, as evidenced by its interaction with PSD95 (Lee et al., 2006), a major scaffold in post-synapse, while genetic disruption of SPIN90 causes a dramatic reduction in filamentous actin in dendritic spines (Kim S. M. et al., 2009). However, the precise mechanism by which SPIN90 participates in plasticity and behavior remains to be clarified.

In this study, we examined the role of actin-binding SPIN90 in synaptic plasticity and behavior. We found that mice in which SPIN90 is genetically ablated show a deficit in NMDA receptor dependent LTD (NMDAR-LTD) and behavioral flexibility, but normal NMDA receptor dependent LTP (NMDAR-LTP). Our findings suggest novel roles of SPIN90 in synaptic plasticity and behavioral flexibility.

## Materials and Methods

### Ethics Statement

This study was carried out in accordance with the recommendations of Gwangju Institute of Science and Technology Animal Care and Use Committee (permit number: GIST-2016-37). The protocol was approved by the Gwangju Institute of Science and Technology Animal Care and Use Committee.

### *Spin90*-Knockout (KO) Mice Generation

Generation of *Spin90*-knockout (KO) mice was described as previously (Kim S. M. et al., 2009). In brief, a target vector was designed to embed a pgk-neomycin cassette into the NheI site of exon 4, which lies upstream of *Spin90*. The target vector was transfected into embryonic cells by electroporation, and targeted clones were screened by subsequent PCR and Southern blot analysis. Heterozygous F1 generation mice were backcrossed with mice of C57BL/6J strain through 17 generations. Heterozygotic females and males were mated to produce *Spin90*-homozygous KO embryos. For genotyping, tail DNA of postnatal day 21 (P21) mice were used to perform PCR with appropriate primers that are specific for wild-type or *Spin90*-KO alleles.

### Antibodies and Reagents

The following primary antibodies were used: anti-PSD95 (Cell Signaling, Danvers, MA, USA, #2507), anti-phospho-Ser473-Akt (Cell Signaling, #4060; #4058), anti-pan Akt (Cell signaling, #2920S), anti-phospho-Ser9-GSK3β (Cell Signaling, #5558), anti-GSK3β (Cell Signaling, #9832), anti-caspase-3 (Cell Signaling, #9662S), anti-cleaved caspase-3 (Cell Signaling, #9664S), anti-Protein phosphatase 1 (PP1; Santa Cruz Biotechnology, Dallas, TX, USA, sc-7482), anti-actin (Santa Cruz Biotechnology, sc-1616), anti-phospho-Thr19-PSD95 (Abcam, Cambridge, MA, USA, ab172628), anti-ionotropic-Ser880-glutamate receptor 2 (GluA2; Abcam, ab52180), anti-GluR1 (Calbiochem, San Diego, CA, USA, PC246), anti-GluA2 (EMD Millipore, Darmstadt, Germany, MAB397), anti-phospho-Ser845-GluA1 (EMD-Millipore, 04-1073), anti-GluA1 (EMD Millipore, MAB2263), anti-WFS1 (ProteinTech, Rosemont, IL, USA, #11558-1-AP), anti-α-tubulin (Sigma Aldrich, St. Louis, MO, USA, T6199), anti-GAPDH (generated in our lab), anti-SPIN90 polyclonal and monoclonal antibodies (generated in our lab).

The following reagents were used: NMDA (Tocris, Bristol, United Kingdom, 0114), CNQX (Tocris, 0190), DiI (1,1′-Dioctadecyl-3,3,3′,3′-Tetramethylindocarbocyanine Perchlorate (“DiI”; DiIC_18_(3))) (Invitrogen, Waltham, MA, USA, #D3911). Horseradish peroxidase-conjugated anti-mouse, anti-rabbit and anti-goat secondary antibodies (Jackson Laboratory, Bar Harbor, ME, USA). Alexa Fluor 488-, 555-, 594-, 647-conjugated secondary antibodies and anti-rabbit IgG, and anti-mouse IgG (Invitrogen, Carlsbad, CA, USA). Harris hematoxylin solution (Sigma Aldrich, HHS32).

### Cell Culture, Homogenization of Brain Tissue and Immunocytochemistry

Cell line culture was performed as described previously (Cho et al., 2013b). HeLa cells were cultured in Dulbecco’s modified Eagle’s medium (DMEM; Gibco, Waltham, MA, USA, 12100-046) supplemented with 10% fetal bovine serum. Primary neuronal cultures were performed as described previously (Cho et al., 2013b). Briefly, hippocampi from wildtype (WT) and *Spin90*-KO embryonic mice (E18–19) were extracted and cultured in neurobasal medium (Invitrogen, Waltham, MA, USA) supplemented with B27 (Gibco, 10889038) and 2 mM GlutaMAX (Gibco, 35050061). Poly-D-lysine-coated coverslips were used to attach cells at a density of 3 × 10^5^ cells/60 mm dish. Eighteen to twenty-one days *in vitro* (DIV) cultured hippocampal neurons were used for cell imaging and biochemical experiments. WT and *Spin90*-KO brain homogenization was adapted from previously described procedures (Cho et al., 2013b). Four-week-old mice whole brains were homogenized in buffered sucrose containing 0.32 M sucrose, 4 mM HEPES (pH 7.4), 1 mM MgCl_2_, 0.5 mM CaCl_2_, 10 mM NaF and 1 mM Na_3_VO_4_ supplemented with protease inhibitor cocktails (Roche, Basel, Switzerland, 04-693-132-001). Lysates were cleared by centrifugation at 4°C (1000 *g* to remove debris, then 12,000 rpm to collect cytosolic fractions). To determine the effects of *Spin90*-KO on the accumulation of PSD95 in dendritic spines and excitatory synapses, neurons were harvested at DIV 18–21. Cells were fixed with 4% paraformaldehyde (PFA) and 4% sucrose in PBS, permeabilized with 0.25% Triton X-100. Fixed cells were incubated with appropriate primary antibodies at room temperature followed by incubation with Alexa Fluor-conjugated secondary antibodies. F-actin and 4′,6-Diamidine-2′-phenylindole dihydrochloride (DAPI) were stained with Alexa Fluor 488-coupled phalloidin for 90 min and 25 min at room temperature, respectively. Images were collected using a Fluoview FV 1000 confocal laser-scanning microscope with 60× oil-immersion objective and images were zoomed 3–4× to acquire image inlets.

### Preparation and Infection of Adeno-Associated Virus (AAV)

6xMyc-tagged hSPIN90 (human) was amplified by PCR using the following specific primers: (sense, 5′-CTAGCT AGCGCCACCATGGAGCAAAAGCTCATT-3′; antisense, 5′-CCCAAGCTTCTAGCTGGGAGCCTCCCCCAGCAC-3′). The amplification products were inserted into pAAV-hsyn-WPRE vectors using NheI and HinDIII restriction enzymes. Adeno-associated virus (AAV; serotype 1) packaging has been performed as described previously (Choi et al., 2014). Briefly, HEK293T cells were transfected with AAV-hsyn-Myc-*SPIN90*, p5E18-RXC1 and pAd-deltaF6. Three days after transfection, AAVs were purified by using iodixanol gradients (60%, 40%, 25%, 15%). After centrifugation (69,000 rpm, 1 h), the 40% layer containing AAV particles was collected. The collected layer was washed with PBS and filtered with an Amicon ultra-15 filter tube (EMD-Millipore, UFC910024). The titer of purified viral vectors was 5.3766 × 10^13^ particles/ml (ppm) as determined by quantitative real-time PCR. Viral titers for infection were determined by infecting DIV10–11 cultured hippocampal neurons with a multiplicity of infection (MOI) of 1000, 2500 or 5000. Neurons were maintained in neurobasal medium supplemented by B27, and no media changes were made after infection. Infected neurons were harvested or fixed at DIV18–20 for appropriate biochemical and immunofluorescent experiments.

### Immunoprecipitation and Immunoblot Analysis

Immunoprecipitation and immunoblot analyses were performed as described previously (Cho et al., 2013b). Briefly, cells were washed with cold PBS and lysed for 1 h at 4°C with modified radioactive immunoprecipitation assay (RIPA) buffer (50 mM Tris-HCl, pH 7.4, 150 mM NaCl, 1% Nonidet P-40 (NP-40), 0.25% sodium deoxycholate, 10 mM NaF, and 1 mM Na3VO4) supplemented with EDTA-free protease inhibitor cocktails (Roche). Lysates were then centrifugated for 10 min at 12,000 rpm, and subjected to Bradford assay (Bio-Rad Laboratories, Hercules, CA, USA, 500-0006) to determine protein concentrations within supernatants. Quantified protein lysates were incubated with appropriate primary antibodies overnight at 4°C, followed by a 4 h incubation with protein A/G sepharose 4 fast flow beads (GE Healthcare, Uppsala, Sweden, 17-0618 for protein A; 17-5280-01 for protein G). Immunoprecipitates were then washed copiously with modified RIPA buffer to quench non-specific binding, subjected to SDS-PAGE and transferred to PVDF membranes. The membranes were blocked with 5% BSA in buffer containing 10 mM Tris-HCl, pH 7.5, 100 mM NaCl and 0.1% Tween 20 or 4% skim milk for detection of phosphorylated or non-phosphorylated proteins, respectively. The membranes were incubated with primary antibodies at 4°C overnight, followed by 1 h incubation of horseradish peroxidase-conjugated secondary antibody at RT, and bands were detected with enhanced chemiluminescence reagent (Dogen, Seoul, Republic of Korea, DLS-1707) and LAS-2000 (Fujifilm, Tokyo, Japan).

### Histology and Diolistic (DiI) Labeling of Brain Slices

Histology experiments were adapted from previous studies (Huh et al., 2013). For staining of hippocampal slices, mice brain were fixed in 10% neutral buffered formalin or 4% PFA, embedded in paraffin, and sectioned at a thickness of 4–5 μm. Paraffin sections were deparaffinized in xylene or Histoclear (National diagnostics, Atlanta, GA, USA, HS-200), dehydrated with ethanol, and stained using hematoxylin and eosin (H&E) or cresyl violet according to the manufacturer’s instructions. Structural patterns in the hippocampus, especially the CA1, CA3, DG and SC regions were evaluated. Stained samples were analyzed using Aperio ImageScope (Leica Biosystems, Wetzlar, Germany). Fluorescent labeling of lightly fixed brian slices using lipophilic dye was adapted from previous studies (Kim et al., 2007; Mahmmoud et al., 2015). For DiI (1,1′-Dioctadecyl-3,3,3′,3′-Tetramethylindocarbocyanine Perchlorate; Life Technologies Corporation, Eugene, OR, USA, D3911) labeling of hippocampal slices, whole brains from 4-week-old male WT or *Spin90*-KO mice were lightly fixed in 1.5% PFA in 0.1 M sodium phosphate buffer for 1 h. One-hundred and fifty micrometer serial coronal hippocampal slices were collected with vibratome (Leica Biosystems, Wetzlar, Germany, VT1000S). DiI was microinjected into CA1 pyramidal layers of hippocampi. After incubation for 16 h, slices were post-fixed in 1.5% PFA for 30 min and counterstained with DAPI. All sections were visualized by Olympus FV1000 confocal microscope and analyzed with Fluoview software.

### Hippocampal Slice Preparation and Electrophysiology

Hippocampal slice preparation and extracellular recordings were performed as described previously (Volk et al., 2010). Briefly, young (3–4 weeks) *Spin90*-KO and WT littermate mice were anesthetized by inhalation of isoflurane dropped onto tissues (250 μl). Transverse hippocampal slices of 300 μm thickness were prepared from each genotype using vibratome (Leica Biosystems, VT1000S). Hippocampal regions were surgically extracted from slices and incubated in cold oxygenated (95% O_2_/5% CO_2_) artificial cerebrospinal fluid (aCSF) containing 125 mM NaCl, 3.25 mM KCl, 25 mM NaHCO_3_, 1.25 mM NaH_2_PO_4_·H_2_O, 11 mM glucose, 0.5 mM CaCl_2_, 5 mM MgCl_2_ for at least 1 h to equilibrate, after which one to two fresh slices were transferred to the recording chamber.

Extracellular field recordings were conducted on an infrared differential interference contrast (IR-DIC) microscope (Nikon E600FN, 2–10× objectives). A truncation was made between the CA3 and CA1 region of the slice before each recording to prevent recurrent activity. Extracellular field recordings were conditioned at 35°C for LTP and 30°C for LTD in aCSF supplemented with 2.5 mM CaCl_2_, 1.5 mM MgCl_2_ and 0 μM picrotoxin (PTX). Field excitatory postsynaptic potentials (fEPSPs) were induced by a 125 μm metal concentric bipolar electrode (FHC) positioned on the stratum radiatum of CA1, and a glass recording electrode (1–2 MΩ) filled with aCSF was placed in its vicinity (~200–400 μm away). Stimulating current was delivered at 30 s intervals from the constant current iso-stimulator (SC-100, WECO). fEPSP was collected by Dagan amplifier (EX-1), filtered at 2 kHz and stored in hard disk of PC.

To determine basal synaptic transmission for each slice, an input-output (I/O) relationship was measured at six stimulation current intensities for each slice through a metal concentric bipolar electrode. As a presynaptic input parameter, fiber volley amplitude was measured as a difference between the baseline and the peak of fiber volley. As a postsynaptic response parameter, the slope of early fEPSP (10%–70% of peak) response was measured. A stimulus intensity producing 35%–55% of maximum postsynaptic response was chosen for the experiment. Maximum response was indicated by the appearance of population spike. The resulting I/O curve was obtained by plotting the fEPSP slope (V/s) vs. fiber volley amplitude (μV). The slope value from linear regression analysis on an I/O plot of each slice was calculated. Next, the magnitude of presynaptic vesicle release probability was assessed by paired-pulse facilitation (PPF) analysis. PPF ratio (fEPSP slope2/fEPSP slope1) was measured by delivering two stimulating pulses at the following intervals (ms); 30, 50, 100, 150, 250 and plotted against paired-pulse interval. For field LTP and LTD experiments, 30 min of baseline recording was made before stimuli for LTP or LTD induction were applied. LTP was evoked by four trains of high-frequency stimulation (4× HFS, 100 Hz for 1 s with 30 s inter-train interval). LTD was induced by low-frequency stimulation (LFS, 1 Hz, 900 single pulses). All long-term plasticity experiments were recorded up to ~100 min. The data were compiled in Excel and plotted using Origin (Microcal Software Inc., Northhampton, MA, USA). Data are presented as responses averaged at 1 min. When average data were plotted, measurements were normalized to the average of baseline responses unless stated otherwise. Data acquisition and analysis were performed by the custom software written in Axobasic 3.1 (Axon Instruments, Sunnyvale, CA, USA).

### Biotinylation and Isolation of the Surface GluA2 Pool

For isolation of synaptic surface GluA2, DIV18–21 cultured hippocampal neurons were incubated in 1 mg/ml EZ-link-NHS-biotin (Thermo Fisher, Waltham, MA, USA) for 20 min at 4°C, washed in PBS twice, then lysed with modified RIPA buffer. Harvested lysates were centrifuged at 14,000 *g* for 15 min at 4°C. Supernatant was removed and 200 μg of protein (GluA2) was mixed with 40 μl of Avidin beads by rotating for 4 h at 4°C. Biotinylated proteins were eluted and blotted. Drug treatments used were as follows: NMDA (100 μM for 5 min, then washout for 15 min) for chemical LTD induction; Na_3_VO_4_ (1 mM for 15 min) and NaF (1 mM for 15 min) in neurobasal media for protein phosphotyrosyl and phosphoseryl phosphatase (PTP) inhibition, respectively.

Isolation of extrasynaptic surface GluA2 was adapted from previous studies (Leonoudakis et al., 2008). Biotinylation was performed as above, then proteins were lysed in modified RIPA buffer supplemented with only 1% NP-40 (or 0.1% Triton-X 100) to minimize PSD solubilization. After incubation at 4°C for 1 h, the lysate was centrifuged at 100,000× *g* for 30 min to pellet the insoluble fraction (enriched in synaptic surface GluA2). The resulting supernatant (enriched in extrasynaptic GluA2) was processed with avidin beads as above.

### Statistical Analysis

Statistical analyses for multiple experiments were conducted based on previous studies (Bae et al., 2012; Cho et al., 2013b). At minimum, three independent experiments were carried out for all data collection. The statistical significance of differences between means was quantified using unpaired Student’s *t*-tests, unless stated otherwise. Data are presented as means ± SEM. To evaluate the distribution of PSD95 within spine heads, fluorescence intensities were analyzed as ratios of the average fluorescence intensities of GFP and PSD95 puncta. PSD95 intensity in spines was determined as the PSD95 intensity in spines with both PSD95- and GFP-positive puncta. The measurements were analyzed using MetaMorph imaging software (Metamorph Inc., Nashville, TN, USA). For spine width/length analysis, approximately 700 spines (from 10 to 14 neurons) at least 100 μm away from soma were selected and measured for each experimental condition. Spines of all subtypes were used in analysis. Spine head width was measured by taking the longest width of the spine head region lying perpendicular to the spine neck axis. Spine head length was measured by the distance between the tip of the spine head to the start of the spine neck, where it meets the dendritic shaft. For spine density analysis in DIV18 cultured neurons and 4-week-old brain slices of both genotypes, individual dendrites were randomly chosen for pyramidal neurons, at least 100 μm away from the soma, and a 10 μm segment was drawn across portions of dendrites containing spine heads, then analyzed with Metamorph software, including all individual spine subtypes. Spine density was calculated by dividing the total number of spines per 10 μm length of dendrite. Individual spine dimensions were grouped initially then normalized per neuron for each condition. For electrophysiological data, average values are expressed as mean ± SEM. Error bars represent SEM. Two-tailed unpaired Student’s *t* test was used at *p* < 0.05 to determine statistical significance.

### Behavioral Test Protocols

#### Open Field Test

An acrylic box (32 cm × 32 cm × 32 cm) was used as an arena. After 30 min of acclimation in the testing room, each mouse was released into the box and allowed to freely explore the arena for 15 min. Total movements in the arena were recorded and analyzed using software (Ethovision XT, Noldus Information Technology, Wageningen, Netherlands). Experimenter was blinded to genotypes of the animals for all the behavioral tests.

#### Rotarod

Rotarod test was performed as previously described (Kang et al., 2015). In brief, each mouse was acclimated to the apparatus for 60 s before the initial training. Then, mice were trained on the accelerated rotarod (4–40 rpm over 300 s). Training was composed of four trials (inter-training interval, 5 min). The times at which mice either fell off from the rotarod or made two complete rotations hanging on the rotarod were recorded.

#### Object-Place Recognition

Object place-recognition test was performed as previously described (Lee et al., 2014). We used an acrylic box (32 cm × 32 cm × 32 cm) as an arena. Two sides of the box had white, opaque walls, and other sides remained transparent. One of the opaque sides had a triangle pattern as a local cue. After 2 days of habituation in the arena for 15 min, mice were released into the box with two identical objects, placed side by side, and allowed to freely explore the objects for 10 min. In test sessions, mice were re-exposed to the same objects for 5 min, but one of which is relocated. Total movements were recorded, and exploration time was scored by experimenter manually. Watching the object within 1 cm, sniffing the object, and touching the object with front paw were considered to be exploration.

#### Social Interaction Test

Social interaction test was performed as previously described (Kang et al., 2015). Six-week-old C57BL/6NSamTac male mice were used as strangers, and a box composed of three compartments isolated by transparent acrylic walls with a small hole was used as arena. On day 1 and day 2, mice were released into the empty arena and habituated to the arena for 15 min. On day 3, a cup with a stranger mouse and another cup with an inanimate object were placed in each side of the compartment. Mice were allowed to freely explore either the stranger or the object for 10 min. Total movements were recorded, and exploration time was scored by experimenter manually.

#### Contextual Fear Conditioning and Memory Extinction

Each mouse was trained with three pairs of a tone (2800 Hz, 85 dB, 30 s) and a co-terminating scrambled foot shock (2 s, 0.4 mA) in a conditioning chamber (Coulbourn Instruments, Whitehall, PA, USA). After training, mice were re-exposed to the same conditioning chamber without any tone or foot shock for six consecutive days. Each re-exposure was separated by 24 h. Auditory fear memory was tested in a novel context, which is composed of a triangle-shaped acrylic insertion, striped wall paper and cleaning agent. The percentage of freezing behavior was scored by using an automated software (Freeze Frame, ActiMetrics, Wilmette, IL, USA).

#### Barnes Maze Test

A white, circular disk (90 cm in diameter) with 20 equally spaced holes (7 cm diameter) located 5 cm from the edge was used as an arena. A black acrylic escape box (15 × 7 × 7 cm) was placed under one hole. Spatial cue with triangle-shaped pattern was placed on one side of the wall in the testing room. Four lights of 60 W were turned on during the trial. One day before the initial training, each mouse was habituated in the escape box for 2 min. The training sessions were repeated for X consecutive days, and X trials per day were separated by 30 min. Mice were placed in an acrylic holding chamber located in the center of the arena before the beginning of each trial. After 10 s of holding, mice were allowed to freely explore the arena and find the escape box for 3 min. During the probe test, mice were released into the maze in the absence of the escape box and the mice were allowed to search the target hole for 90 s. During reversal training, the location of the target hole was relocated to the opposite side of the circular maze from its location in initial training. The training procedure for the reversal training was same as the initial training, and sessions were repeated for four consecutive days, and three trials per day. All movements were recorded, and durations in each zone were analyzed using Ethovision XT software (Noldus Information Technology, Wageningen, Netherlands).

## Results

### Structural Disorganization of CA1 Neurons in *Spin90*-KO Hippocampus

**Figure 1:**
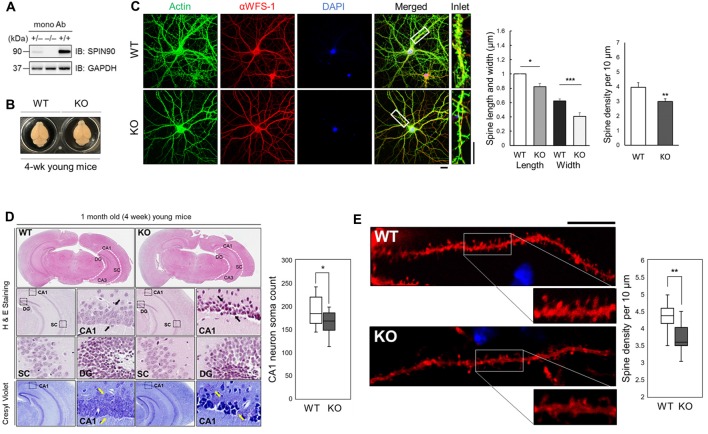
SH3 protein interacting with Nck 90 kDa *(Spin90)* deletion disrupts spine morphology and CA1 hippocampal structure. **(A)** SPIN90 expression in wild-type (+/+), heterozygous (+/−) and knockout (KO) (−/−) homozygous mice. Western blot analysis of brain tissues from each genotype detected with monoclonal SPIN90 antibody. **(B)** Exterior gross morphology of wildtype (WT) and *Spin90*-KO mice brains fixed in 1.5% paraformaldehyde (PFA). **(C)** Immunocytochemistry of DIV18–21 cultured hippocampal neurons stained by CA1 neuron marker, αWFS1 (red), phalloidin (green) and DAPI (blue). Scale bars represent 20 μm (short) and 10 μm (long). Statistical analysis of spine length and width (in μm) (*n* = 27–33) and spine density (*n* = 36–44). **(D)** Histological assessment of WT and *Spin90*-KO hippocampal slices by H&E and cresyl violet staining. CA3, CA1, DG, SC subregions were qualitatively assessed. Black arrows indicate structural differences in soma distribution in each region of the hippocampus. Yellow arrows indicate loss of soma in CA1. Quantitative cell count of CA1 soma analysis (*n* = 13 for both genotypes). **(E)** Spine density analysis in CA1 neurons of DiI labeled WT or *Spin90*-KO hippocampal slices. Spine density was determined by dividing the number of spines per 10 μm dendrite (*n* = 49 for WT, *n* = 54 dendritic segments for *Spin90*-KO). Scale bar and white box length are 10 μm. All data are expressed as means ± SEM (*p < 0.05; **p < 0.01; ***p < 0.001).

Previous studies have revealed structural alterations in dendritic spines of *Spin90*-KO cultured hippocampal neurons (Kim S. M. et al., 2009). To gain better insight into SPIN90 function *in vivo*, we further delineated the mechanisms underlying SPIN90 loss-of-function in mouse tissue. *Spin90*-KO mice were generated as previously described (Kim S. M. et al., 2009). Complete depletion of *Spin90* in young (4-week-old) homozygous *Spin90*-KO (−/−) mice compared with WT (+/+) controls and heterozygous (+/−) mice was confirmed by immunoblotting of forebrain fractions with a monoclonal anti-SPIN90 antibody (Figure 1A). Brain size did not vary between WT and *Spin90-KO* mice, and no significant irregularities in exterior gross morphology were found (Figure 1B). Significant reduction in spine width, length and density were confirmed in *Spin90*-KO hippocampal neurons (DIV 18) compared with WT neurons, both immunostained with CA1 neuron marker αWFS1 and F-actin-binding phalloidin (Figure 1C). Histological analyses revealed correct anatomical distribution of neurons in the CA3 (*cornu Ammonis area* 3) and DG (dentate gyrus) regions of the hippocampus, but showed structural disorganization and significantly reduced soma number in the CA1 (*cornu Ammonis area* 1) stratum radiatum in *Spin90*-KO slices compared with those from WT mice (Figure 1D). Moreover, spine density was significantly reduced in CA1 neurons of *Spin90*-KO hippocampal slices compared with WT (Figure 1E and Supplementary Figure S1). Together, these observations demonstrate that SPIN90 innately regulates structural organization and spine density in hippocampal CA1 neurons and may possibly affect the overall output strength of the CA1 circuitry in the hippocampus by regulating the number of the CA1 pyramidal neuron.

### NMDA Receptor-Dependent LTD Is Impaired in *Spin90*-KO Hippocampal Slices

**Figure 2:**
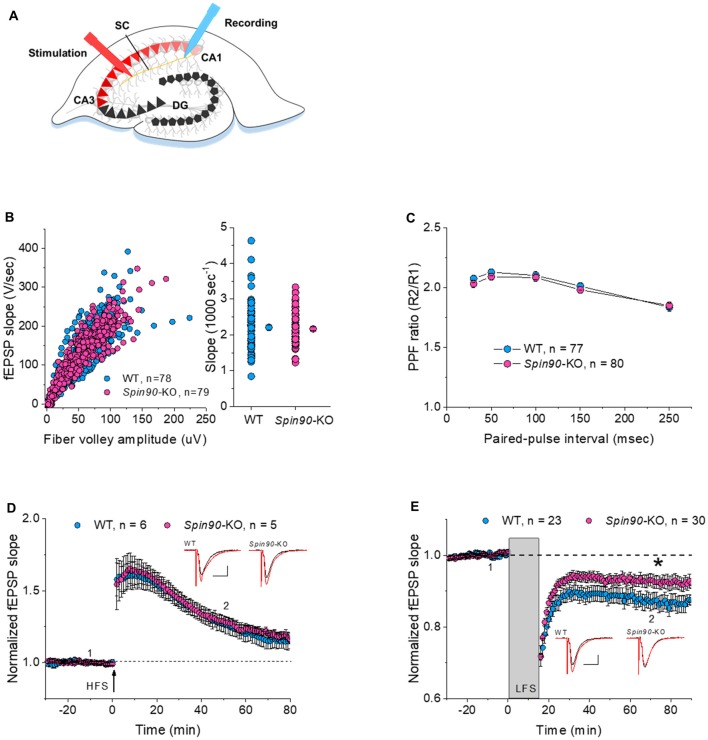
Normal synaptic transmission and long-term potentiation (LTP), but impaired long-term depression (LTD), in *Spin90*-KO mice. **(A)** Schematic diagram of extracellular field recordings on WT (+/+) and *Spin90*-KO (−/−) hippocampi. CA3 region of slices was stimulated by electrodes, and signals propagated through the Schaffer Collateral (SC) axons to the CA1 region were recorded as EPSPs. **(B)** Synaptic input-output relationship obtained by plotting the slopes of evoked field excitatory postsynaptic potentials (fEPSPs) against fiber-volley amplitude (*n* = 78 for WT, *n* = 79 for *Spin90*-KO). **(C)** Ratios of paired-pulse facilitation (PPF) plotted as a function of paired-pulse interval (*n* = 77 for WT, *n* = 80 for *Spin90*-KO). **(D)** Representative traces from individual CA1 neurons following HFS stimulation showing normal N-methyl-D-aspartate receptor (NMDAR)-dependent LTP in *Spin90*-KO neurons. NMDAR-LTP induced by four theta-burst protocol in young CA1 hippocampal slices. The insets are example average traces for 2 min. Baseline trace (black) and LTP trace (red) were collected at 1 and 2, the time points indicating 10 min before and 50 min after LTP induction, respectively. Scale bars, 10 ms and 150 μV (*n* = 6 for WT; *n* = 5 for *Spin90*-KO). **(E)** NMDAR-LTD was induced by low-frequency stimulation (LFS) at CA1 neurons in WT and *Spin90*-KO slices. The insets are example average traces for 2 min. Baseline trace (black) and LTD trace (red) were collected at 1 and 2, the time points indicating 10 min before and 55 min after LTD induction, respectively. Scale bars, 10 ms and 150 μV (*n* = 23 for WT; *n* = 30 for *Spin90*-KO). Data are represented as mean ± SEM (*p < 0.05).

It is well understood that inter-synaptic communication not only affects the formation and development of synapses, but also synaptic plasticity (Yuste and Bonhoeffer, 2001; Citri and Malenka, 2008). As previous studies have shown, the physiological relevance of SPIN90 in plasticity may include synaptic activation, involving N-methyl-D-aspartate receptor (NMDAR), which is closely tied with changes in spine structure (Cho et al., 2013b). Therefore, we next investigated the role of SPIN90 in synaptic plasticity by performing extracellular field recordings in acute hippocampal slices from young (4 weeks old) mice (Figure 2A). An examination of basal synaptic transmission revealed no differences between *Spin90*-KO and WT slices, determined by assessing the input-output relationship between presynaptic fiber volley amplitude (μV) and fEPSP slope (V/s; Figure 2B). Presynaptic vesicle release probability, assessed by paired pulse facilitation (PPF) ratios, was not significantly different between the two genotypes (Figure 2C). Thus, we next addressed postsynaptic responses at Schaffer collateral-CA1 synapses, using NMDAR-dependent LTP and LTD protocols. We first tested conventional LTP induced by a burst of high-frequency stimulation (4× HFS, 100 Hz), which resulted in robust synaptic potentiation in both WT and *Spin90*-KO slices, gradually decaying to baseline in ~70 min (Figure 2D). Next, NMDAR-dependent LTD (NMDAR-LTD) was induced by a long train of LFS (15 min at 1 Hz), which resulted in robust synaptic depression in WT slices following a persistent plateau at ~70 min. However, the induction of LTD was mildly but significantly impaired in *Spin90*-KO slices (Figure 2E), indicating a deficit in maintenance of LTD. This difference was not due to changes in basal synaptic transmission or presynaptic function between *Spin90*-KO and WT mice, as input-output relationships for each group of mice in the slope of the fEPSP, and presynaptic fiber volley against PPFs were similar in magnitude. Collectively, these results suggest that SPIN90 deficiency in CA1 pyramidal neurons primarily affected the activity-dependent processes of LTD, but not basal homeostatic synaptic transmission of CA1 neurons of the hippocampus.

### AMPAR Endocytosis Is Attenuated in the Absence of SPIN90

**Figure 3:**
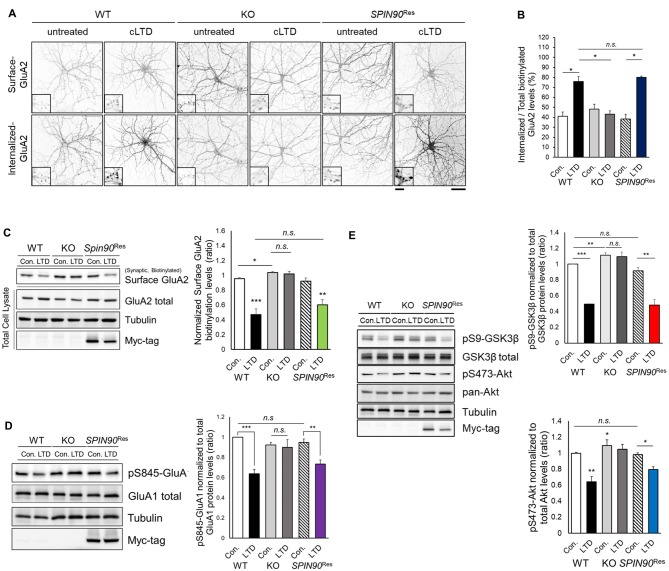
Chemical forms of LTD (cLTD)-induced AMPAR endocytosis is blocked in *Spin90*-KO neurons. **(A)** AMPAR internalization assay on DIV18 hippocampal neurons. Surface GluA2 levels indicate constitutive AMPAR trafficking and internalized levels are those endocytosed after cLTD induction (100 μM NMDA application for 5 min and 15 min wash). Scale bar, 5 μm and 20 μm. **(B)** Internalized GluA2 levels were plotted against total biotinylated GluA2 and quantified (*n* = 8–15 for WT and *Spin90*-KO; *n* = 3 for adeno-associated virus (AAV)-6xMyc-*SPIN90* infected *Spin90*-KO neurons (*SPIN90*^Res^). **(C)** Immunoblot analysis of surface biotinylated GluA2 levels after cLTD induction in WT, *Spin90*-KO and *SPIN90*^Res^ neurons (*n* = 4 for all genotypes). **(D)** Immunoblot analysis of dephosphorylation of Ser845-GluA1 after cLTD induction (*n* = 9 for WT and *Spin90*-KO; *n* = 4 for *SPIN90*^Res^). **(E)** Changes in phosphorylation of Akt-GSK3β signaling upon cLTD induction (*n* = 14 for WT and *Spin90*-KO; *n* = 4 for *SPIN90*^Res^). All data are expressed as mean ± SEM (*p < 0.05, **p < 0.01, ***p < 0.001, *n.s*., non-significant).

NMDAR-LTD induction requires AMPAR endocytosis, which is mediated by phosphatase-dependent AMPAR dephosphorylation (Seo et al., 2014). Considering *Spin90*-KO slices show deficit in LTD, we reasoned that SPIN90 might play a role in LTD by facilitating AMPAR endocytosis. Since chemical forms of LTD (cLTD) induced by NMDA has been known to share a mutual mechanism with homosynaptic NMDAR-dependent LTD, we induced cLTD on DIV18–21 primary cultured hippocampal neurons (Lee et al., 2000). We first determined the basal expressions of the AMPAR subtypes, GluA1 and GluA2, in *Spin90*-KO and WT hippocampal neurons. Surface expression of GluA2 but not GluA1, determined by biotinylation, was modestly but significantly increased in *Spin90*-KO compared with WT (Figure 3C and Supplementary Figure S2A). Next, cLTD experiments were performed as previously described (Cho et al., 2013b). During induction of cLTD by treatment of DIV18–21 cultured hippocampal neurons with NMDA (5 min, 100 μM), internalized GluA2 levels were markedly increased in WT neurons, but no such increase was observed in *Spin90*-KO neurons (Figures 3A,B). To further validate whether these effects were specific to SPIN90 deficiency, we performed an autonomous rescue of SPIN90 in cultured neurons using recombinant AAV-6xMyc-hSPIN90 (AAV-6xMyc-*SPIN90*; Supplementary Figure S2B). Indeed, internalization of biotinylated-GluA2 was recovered in AAV-6xMyc*-SPIN90* infected *Spin90*-KO neurons (SPIN90 rescued, *SPIN90*^Res^; Figures 3A,B). In addition, surface levels of GluA2 were nearly unchanged in *Spin90*-KO neurons after cLTD induction, which was also reversed in SPIN90 rescued neurons, as confirmed by immunoblot analysis (Figure 3C). By contrast, extrasynaptic surface GluA2 levels were readily reduced in all WT, *Spin90*-KO and *SPIN90*^Res^ groups (Supplementary Figure S2C), which indicated that activity-dependent internalization of extrasynaptic GluA2 was normal in *Spin90*-KO neurons and therefore suggests that GluA2 internalization defect in *Spin90*-KO neurons was specific to synaptic membrane GluA2.

Because AMPAR endocytosis is followed by dephosphorylation of GluA1 at Ser845 (Lee et al., 2000), we tested GluA1 phosphorylation change in *Spin90*-KO neurons during cLTD induction. cLTD induced a robust reduction (~30%) in GluA1 phosphorylation at Ser845 in WT neurons, whereas these changes were not detected in *Spin90*-KO neurons following cLTD (Figure 3D). Both biotinylated surface GluA2 and S845-GluA1 dephosphorylation after cLTD induction were rescued in *SPIN90*^Res^ neurons (Figures 3C,D). These data indicate that SPIN90 deficiency negatively regulates trafficking of AMPARs under cLTD induction.

To further investigate the mechanisms underlying deficits in LTD and AMPAR internalization, we focused on glycogen synthase kinase 3 beta (GSK3β), a kinase known to mediate AMPAR dephosphorylation during LTD induction (Kim et al., 2011). GSK3β is known to phosphorylate the T19 residue of PSD95, that acts as a pre-requisite for triggering AMPAR endocytosis and LTD (Nelson et al., 2013). Akt negatively regulates GSK3β activity as an upstream inhibitor, thereby blocking LTD induction (Peineau et al., 2007). We hypothesized that dysfunction in Akt-GSK3β signaling might be key to the impairments exhibited by *Spin90*-KO neurons. Normally, serine 9 (Ser9) of GSK3β is dephosphorylated upon LTD induction, resulting in kinase activation. We observed LTD induction significantly decreased the phosphorylation of Ser9-GSK3β in WT neurons, which was absent in *Spin90*-KO neurons (Figure 3E), suggesting that cLTD failed to activate GSK3β signaling in KO neurons.

Interestingly, GSK3β phosphorylation levels were elevated in both untreated and cLTD-induced *Spin90*-KO neurons, indicating significant suppression of GSK3β activity in the absence of SPIN90 (Figure 3E). Further evidence for suppression of GSK3β activity was provided by an examination of the levels of Ser473-phosphorylated Akt, which showed that LTD-induced Akt dephosphorylation was reduced to a lesser degree in *Spin90*-KO neurons than in WT neurons (Figure 3E). Thus, the deletion of SPIN90 significantly increased the phosphorylated (inactive) form of GSK3β in the resting state, which might be mediated by dysregulation of Akt. Moreover, reintroduction of SPIN90 into *Spin90*-KO neurons via AAV infection rescued Akt-GSK3β signaling, indicating that SPIN90 was sufficient to induce AMPAR endocytosis and LTD, while excluding the possibility that the effects observed in *Spin90*-KO neurons were due to secondary changes (Figure 3E). Taken together, these results considerably suggest a novel physiological function for SPIN90 in AMPAR endocytosis that is likely mediated through its inhibition of Akt-GSK3β signaling in response to neuronal activation.

### Impaired Akt-GSK3 Signaling in *Spin90*-KO Neurons Is Attributable to Akt-SPIN90 Interactions

**Figure 4:**
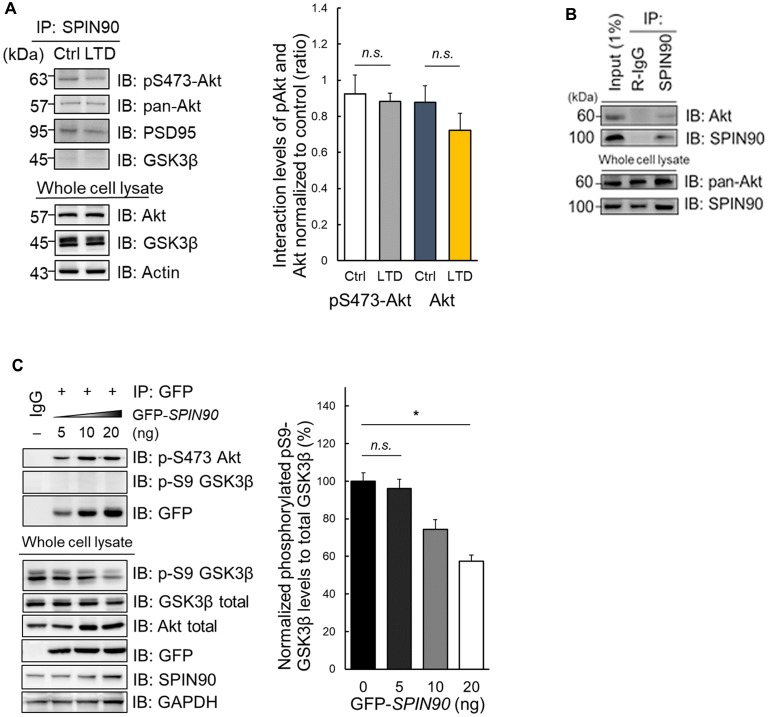
Impaired Akt-GSK3β signaling in *Spin90*-KO neurons is due to Akt-SPIN90 interaction. **(A)** DIV21 WT hippocampal neurons were treated with or without cLTD and lysates were harvested for immunoprecipitation with anti-SPIN90 antibody. SPIN90 interaction with pSer473-Akt, pan-Akt and GSK3β were assessed. **(B)** HeLa cells were harvested and immunoprecipitated with anti-SPIN90 antibody. Akt and SPIN90 binding was assessed. **(C)** Dose-dependent overexpression of GFP-*SPIN90* in HeLa cells followed by immunoprecipitation with either pSer473-Akt, pSer9-GSK3β and GFP(SPIN90). pSer9-GSK3β activity was measured *in vitro* by a decrease in phosphorylation levels as dose of GFP-SPIN90 transfection reached from 5 ng to 20 ng. Data are expressed as mean ± SEM (*p < 0.05; *n.s*., non-significant).

During LTD induction, Akt is dephosphorylated at Ser473, enabling GSK3β activation (Bradley et al., 2012). Akt also binds to and participates in the regulation of Girdin, an ABP that is crucial for plasticity and learning (Nakai et al., 2014). This led us to investigate whether another ABP SPIN90 may also bind to Akt, which subsequently plays a role in LTD. Indeed, we found that SPIN90 interacts with both phosphorylated Akt at Ser473 and total (pan) Akt in cultured neurons (Figure 4A). Moreover, we found no significant changes in SPIN90 interaction with pSer473-Akt and pan Akt after cLTD induction (Figure 4A). Similar interactions of pan-Akt with SPIN90 were also confirmed in HeLa cells (Figure 4B). To determine how Akt binding to SPIN90 affects GSK3β activation, we variably overexpressed GFP-*SPIN90* (5–20 ng expression plasmid) in HeLa cells and co-immunoprecipitated pSer473-Akt (Figure 4C). These experiments showed a gradual increase in SPIN90 binding to immunoprecipitated pSer473-Akt, determined by probing for GFP. pSer9-GSK3β phosphorylation decreased with increasing overexpression of SPIN90, reaching a maximum reduction of ~50% (Figure 4C). These data suggest that Akt binds to SPIN90, which may serve as an adapter to inhibit Akt from phosphorylating its effector, GSK3β. It appeared that the absence of SPIN90 removes such sequestration of Akt, thereby allowing GSK3β phosphorylation and inactivation intrinsically.

### PSD95 Dissociation Is Blocked during cLTD of *Spin90*-KO Cultured Neurons

**Figure 5:**
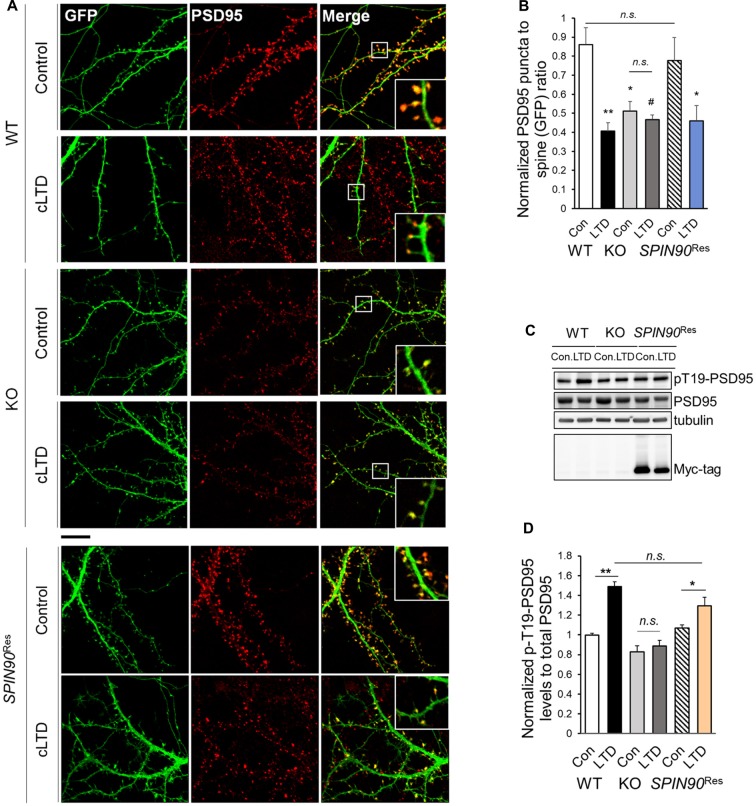
SPIN90 is necessary for PSD95 dissociation during cLTD. **(A)** DIV18–21 WT, *Spin90*-KO and AAV-6xMyc-*SPIN90* infected *Spin90*-KO neurons (*SPIN90*^Res^) hippocampal neurons expressing GFP and PSD95 were pre-incubated with CNQX (10 μM) for 1 h before cLTD treatment, followed by immunostaining with GFP (green) and PSD95 (red) antibody. Scale bars indicate 5 μm. **(B)** The localization of PSD95 in ratios of PSD95 to GFP puncta was analyzed (*n* = 13–21 for WT and *Spin90*-KO; *n* = 19 for *SPIN90*^Res^-control(Con), *n* = 21 for *SPIN90*^Res^-LTD). **(C)** DIV18 WT, *Spin90*-KO and *SPIN90*^Res^ neurons were subjected to cLTD treatment followed by harvesting of cells and immunoblot analysis. Phosphorylation of PSD95 at Thr19 was assessed. **(D)** Quantification of pThr19-PSD95 levels normalized to total PSD95 after cLTD induction (*n* = 9 for WT and *Spin90*-KO; *n* = 3 for *SPIN90*^Res^). All data are expressed as mean ± SEM (^#^*p* = 0.0629, *p < 0.05, **p < 0.01, *n.s*., non-significant).

It is known that a strong reduction in long-term plasticity characterized by decreased synaptic strength, is correlated with spine shrinkage (Zhou et al., 2004). Accordingly, we determined the effects of SPIN90 ablation on spine morphology in cultured hippocampal neurons with or without cLTD induction. PSD95 immunostaining in WT and *Spin90*-KO neurons were quantified by measuring PSD95 density per spine, normalized to GFP-control. The head size of mushroom spines in WT neurons was dramatically reduced during cLTD, confirming normal spine shrinkage (Figure 5A). By contrast, the head size of spines was significantly decreased in *Spin90-KO* neurons under basal conditions, and remained unaltered even after cLTD induction (Figures 5A,B). When *Spin90*-KO neurons were autonomously rescued by SPIN90 expression, the mushroom spines during basal and spine shrinkage during cLTD conditions were recovered (Figures 5A,B). Spine shrinkage is correlated with disassociation of PSD95 from spine heads to shafts, and this disassociation requires phosphorylation of Thr19 residue of PSD95 (Nelson et al., 2013). Thus, we determined whether Thr19-PSD95 phosphorylation was inhibited in *Spin90*-KO neurons. Administration of NMDA induced a significant increase in Thr19-PSD95 phosphorylation in WT neurons, but no such changes were detected following NMDA treatment in *Spin90*-KO neurons (Figures 5C,D). Whereas, SPIN90 rescue reversed the effects of SPIN90 depletion on Thr19-PSD95 phosphorylation (Figures 5C,D). Collectively, these results indicate that PSD95 may not dissociate from spine heads in *Spin90*-KO neurons; thus spine heads maintain their intrinsic spine size even after LTD induction. Moreover, failure to undergo cLTD-induced spine shrinkage in *Spin90*-KO neurons is attributable, at least in part, to the dysregulation in PSD95 dissociation from spine heads.

### Memory Extinction and Behavioral Flexibility Are Impaired with the Loss of SPIN90

**Figure 6:**
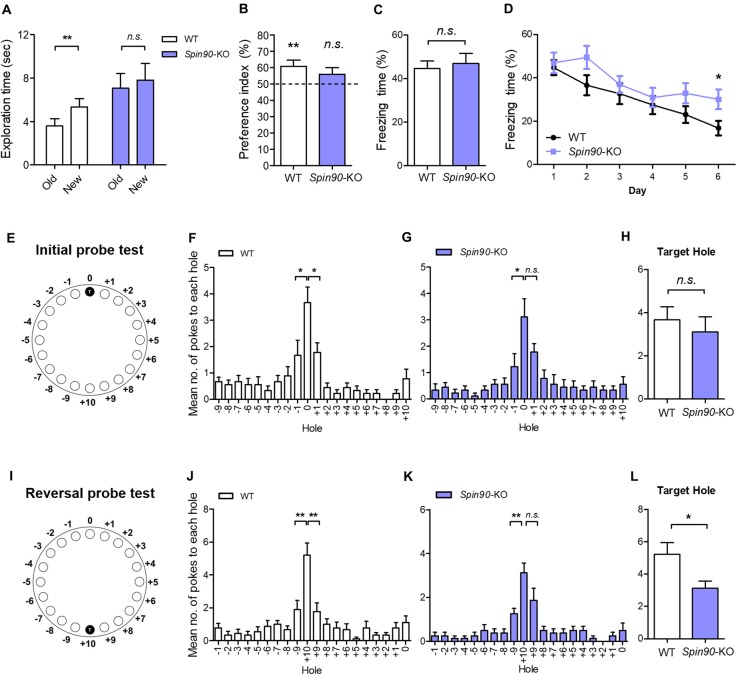
Behavioral flexibility is reduced in *Spin90*-KO mice. **(A,B)** Object-place recognition test. **(A)** Placed novel object is termed “new” vs. the familiar “old”. **(B)** Preference for exploring new object vs. old object is assessed in WT (*n* = 20; **p < 0.01) and *Spin90*-KO mice (*n* = 16; *p* = 0.3819). **(C,D)** Fear conditioning and fear extinction paradigm. Freezing levels indicative of fear memory were assessed on day 1 after training in WT and *Spin90*-KO mice. Extinction tests were performed up to day 6 after training and freezing levels were compared between both genotypes (*n* = 13 for both; *p* = 0.7030, *p < 0.05). **(E–H)** Initial learning in Barnes maze test. **(E)** Position of the target hole during initial learning is marked T (0). **(F)** Probe tests were taken to examine WT and *Spin90*-KO mice ability to find target hole, indicated by number of nose pokes. Mean number of pokes to each hole adjacent to the target hole suggests proper learning was achieved. WT mice (*n* = 9) showed higher number of nose pokes to target (0) hole vs. −1 hole (unpaired *t* test, *p < 0.05) and +1 hole (unpaired *t* test, *p < 0.05). **(G)** *Spin90*-KO mice (*n* = 9) showed higher number of nose pokes to target (0) hole only vs. −1 hole (unpaired *t* test, *p < 0.05). **(H)** No significant differences were found in ability to find target hole (unpaired *t*-test, *n.s*., *p* = 0.5542). **(I–L)** Reversal learning in Barnes maze test. **(I)** Position of the new target hole during reversal learning is marked as T (+10). **(J)** Probe tests were taken to differentiate WT and *Spin90*-KO mice ability to find target hole in new location. WT (*n* = 9) showed higher number of nose pokes to target (+10) hole vs. −9 hole (unpaired *t* test, **p < 0.01) and +9 hole (unpaired *t* test, **p < 0.01). **(K)** *Spin90*-KO (*n* = 8) showed higher number of nose pokes to target (+10) hole only vs. +9 hole (unpaired *t* test, **p < 0.01). **(L)** *Spin90*-KO showed significantly less number of nose poking into the target hole (unpaired *t*-test, *p < 0.05). All data are expressed as mean ± SEM.

It is well understood that structural and functional plasticity are closely tied with behavioral modification such as learning and memory (Lamprecht and LeDoux, 2004). Particularly, in the hippocampus, NMDAR-LTD has been reported to be involved in behavioral flexibility, episodic-like memory, immediate memory of a novel context and novelty-detection in the hippocampus (Kim et al., 2011). Therefore, we investigated whether the deletion of SPIN90 affects any cognitive functions. Basal locomotive activity and anxiety level were comparable between SPIN90 KO and WT controls (Supplementary Figures S3A–F). In addition, *Spin90*-KO mice showed normal social behaviors compared with WT (Supplementary Figures S3G–I). Since SPIN90 deficiency caused NMDA receptor-dependent form of hippocampal synaptic plasticity, we then subjected the KO mice to a series of hippocampus-dependent memory tasks. Firstly, *Spin90*-KO mice were tested in the object-place recognition test. Whereas WT mice spent significantly more time exploring the object at the new location during the test session 24-h after training session, *Spin90*-KO mice failed to distinguish the displaced object from the other object placed at the same location (Figures 6A,B), indicating that object-place recognition memory is impaired in *Spin90*-KO mice. We then performed contextual fear conditioning to assess contextual memory in both genotypes where mice learn to associate the context and an aversive event (mild electric shock; Figures 6C,D). During the context-dependent memory test performed 24 h after training, the freezing behavior of *Spin90*-KO mice was comparable to that of WT mice, indicating normal associative memory formation with SPIN90 deficiency (Figure 6C).

Since NMDAR-dependent LTD impairment has been associated with the deficits in behavioral flexibility (Nicholls et al., 2008; Kim et al., 2011; Mills et al., 2014), we further examined the extinction of fear conditioned memory. Interestingly, *Spin90*-KO mice showed significantly higher levels of freezing behavior on subsequent trials from day 1 to day 6, compared with a marked decay of freezing behavior observed in WT mice, indicating impaired memory extinction (Figure 6D). This pronounced deficit in memory extinction likely reflects the physiological impairment of LTD exhibited by *Spin90*-KO mice (Figure 2E). Elaborating on this result, we further employed the Barnes maze test, which is another hippocampus-dependent task (Barnes, 1979; Figures 6E–L). Although *Spin90*-KO showed longer latency to the target hole in the first day of training, *Spin90*-KO and WT did not show significant difference at the end of initial learning session (Supplementary Figure S4). During the probe trial after initial training trials (Figures 6E–H), WT mice showed significantly higher number of nose poking to the target hole compared to the other adjacent holes (Figure 6F), while *Spin90*-KO mice failed to distinguish the target hole and one of the adjacent holes (Figure 6G). However, WT and *Spin90*-KO mice showed comparable numbers of nose poking at the target hole (Figure 6H), suggesting that the memory deficit in the KO is very mild. We then performed reversal learning by moving the escape box to the opposite side of the maze (Figures 6I–L). Both genotypes equally learned the new target position (Supplementary Figure S4). However, *Spin90*-KO mice exhibited significantly less numbers of target hole poking than WT controls during the reversal probe test (Figures 6K,L), confirming that SPIN90 deficiency may have impaired the ability to adapt flexibly to changed environments. Altogether, these data provide a behavioral role for SPIN90 in hippocampus-dependent learning and behavioral flexibility.

## Discussion

LTD is a form of plasticity that regulates memory processing and provides vacancies to allow new memories to be stored (Nicholls et al., 2008). Interestingly, the maintenance of LTD is blocked by inhibiting actin depolymerization, as evidenced by the finding that long-lasting forms of LTD are associated with a decrease in filamentous F-actin content in spines (Star et al., 2002). Previous studies using neuron cultures have suggested that SPIN90, an ABP is also involved in LTD (Cho et al., 2013a,b). In this study, we investigated the role of SPIN90 in synapse and behavior by using *Spin90*-KO mice.

The cognitive defects exhibited by *Spin90*-KO mice covered a broad range. At one extreme, these mice displayed no anxiety-like behavior in the open-field test or the radial arm maze (Supplementary Figure S3). Locomotor and motor performance also appeared to be normal as assessed by rotarod tests (Supplementary Figure S3). To test memory phenotypes, we performed object-place recognition, contextual fear conditioning, and Barnes maze test, all of which are hippocampus-dependent paradigms (Lamprecht and LeDoux, 2004). *Spin90*-deficient mice showed deficits in object-place recognition memory test and initial learning in the Barnes maze (Figure 6). However, the deficits were very mild and *Spin90*-KO mice showed comparable memory performance to WT littermates in contextual fear conditioning, suggesting that SPIN90 deletion may have limited impact on hippocampus-dependent learning. These results, together with the findings of normal LTP in the *Spin90*-KO slices, indicate that SPIN90 may not be necessary for this form of learning and synaptic plasticity.

Memory extinction has been associated with the deterioration of existing memory, caused by the degradation of memory-related molecules (Seo et al., 2014), and recent studies have suggested that extinction could be a specific phenomenon mediated by intricate molecular interactions during the learning paradigm (Tronson et al., 2012). For example, it has been shown that knock-in mice expressing a mutated form of the Cdk5-activator, p35, that cannot be cleaved to p25 (Δp35KI mice) are prone to impaired fear memory extinction and behavioral flexibility (Seo et al., 2014). In addition, both deletion and pharmacological inhibition of phosphoinositide 3-kinase gamma (PI3Kγ), known to modulate cortical actin density, disrupts NMDAR-LTD while leaving other forms of synaptic plasticity unaffected (Kim et al., 2011). This blockade specifically correlates with deficits in behavioral flexibility, in which residual memory is maintained and reversal learning is impaired.

Thus, based on the blockade of LTD in *Spin90*-KO mice, we proposed that acquired preexisting memories may degrade at a slower rate in these mutant mice. Consistent with previous findings, fear memory extinction was significantly reduced in the absence of SPIN90 (Figure 6D), while leaving memory acquisition intact (Figure 6C). Therefore, we speculated that SPIN90 might be involved in modulating either learned memory extinction or long-term memory retention. These results were further confirmed by the Barnes Maze test, where *Spin90*-KO mice showed memory deficits only after the reversal learning, but not after the initial learning trials (Figures 6E–L), which suggests that behavioral flexibility, the innate ability adapt to environmental circumstances (to spontaneously forget, in this case), has been disrupted in *Spin90*-KO mice. We then explored whether *Spin90*-KO mice are predisposed to autism-like phenotypes such as altered social behavior and hyperactivity, owing to blockade of LTD. However, social memory in *Spin90*-KO mice were comparable to that of WT (Supplementary Figures S3G–I). Thus, SPIN90 is exclusively required for behavioral flexibility and discriminative recognition learning, but has diverging roles in each learning paradigm.

**Figure 7:**
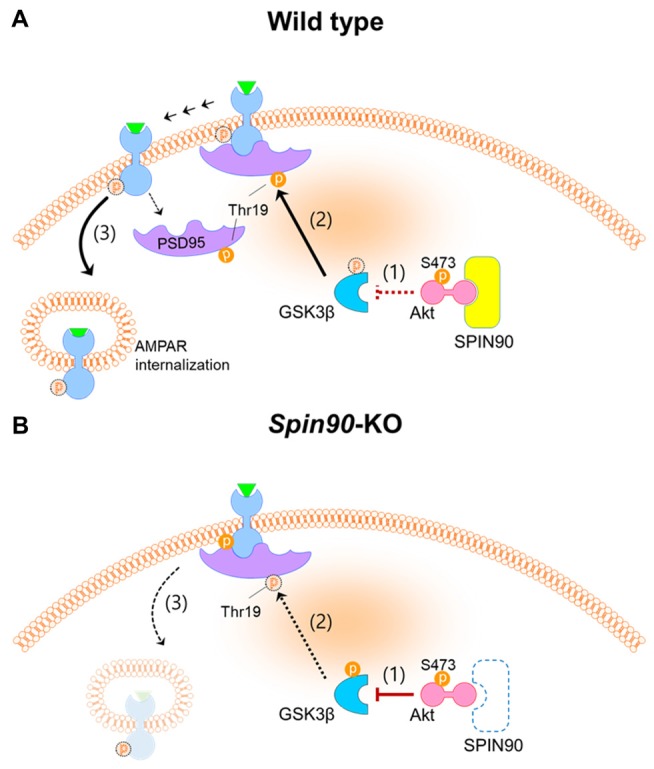
Schematics of SPIN90 during NMDAR-LTD. **(A)** LTD induction in normal (WT) neurons; **(1)** Upon NMDAR stimulation, SPIN90 binds to Akt, sequestering it from phosphorylating downstream GSK3β. **(2)** Active GSK3β is able to phosphorylate PSD95 at Thr19, which causes **(3)** PSD95 to dissociate from GluA2-containing AMPAR, which subsequently induces AMPAR internalization and LTD. **(B)** LTD induction in *Spin90*-KO neurons; **(1)** Absence of SPIN90 allows activated Akt (phosphorylation at Ser473 sequesters GSK3β, thereby **(2)** hindering GSK3β from phosphorylating PSD95. **(3)** GluA2-containing AMPARs remain “docked” on PSD95, unable to internalize. The defect in AMPAR internalization and LTD in *Spin90*-KO neurons may consequently have secluded excess AMPARs on the surface, which is apparent in Figures 3A–C.

It has been well documented that cLTD, induced by brief bath application of NMDA, and electrically stimulated homosynaptic (input-specific) LTD share a mutual expression mechanism both of which correlate with activation of NMDA receptor, leading to AMPAR internalization (Collingridge et al., 2010). Further, we used dissociated cultured neurons, in which stimulation can be induced locally or entirely on the network and because analyses of neurons was more easily achieved after LTD induction than in an *in vivo* environments (Potter and DeMarse, 2001). To investigate the molecular and cellular mechanism underlying LTD deficit in *Spin90*-KO, we performed cLTD experiments on DIV18–21 cultured hippocampal neurons. AMPARs are anchored at synaptic scaffolds such as PSD95 and are unable to internalize into the plasma membrane unless several pre-requisites are met, including GSK3β-mediated phosphorylation of PSD95 at Thr19 (Nelson et al., 2013). PSD95 undocks AMPARs upon phosphorylation and dissociates from the membrane to facilitate AMPAR internalization. Interestingly, expression of SPIN90 is particularly strong at PSDs in the synaptic compartment of spines (Lee et al., 2006). Therefore, we sought to address PSD95 function in the absence of SPIN90. We found that phosphorylation of PSD95 at Thr19 was obstructed in *Spin90*-KO neurons compared with WT neurons, indicating that AMPAR internalization was significantly blocked (Figures 5C,D). We confirmed that LTD in WT neurons shrunk spines by nearly 50% while KO neurons had an intrinsic reduction in spine head size that was not further affected by LTD (Figures 5A,B). Therefore, we concluded that the intrinsic spine shrinkage apparent in *Spin90*-KO neurons may be, dependent, at least in part, on PSD95 dissociation from spine heads (Figure 7).

The spine shrinkage observed during LTD is caused in part by internalization of surface AMPARs into spines through a clathrin-dependent mechanism, but also reflects depolymerization of actin by severing proteins such as cofilin (Zhou et al., 2004). Interestingly, activation of cofilin in *Spin90*-KO neurons was blocked after LTD induction, suggesting that cofilin-mediated spine shrinkage is essentially obstructed in the absence of SPIN90 (Supplementary Figure S5A). This result may not be unexpected, given that we previously identified SPIN90 as a modulator of cofilin-mediated spine shrinkage (Cho et al., 2013b). Further, it has been noted that thinner filopodia-like spines with smaller volume-to-surface ratios may result from less effective internalization of AMPARs upon synaptic input (Matsuzaki et al., 2001). Thus, whether *Spin90*-KO neuron deficiency in LTD is because AMPARs are unable to internalize into already shrunken spine heads or simply because AMPAR internalization signaling is inhibited is a matter of debate. However, recent studies indicated that synaptic depression such as LTD could be uncoupled to spine shrinkage (Zhou et al., 2004).

The decrease in basal PSD95 puncta density in *Spin90*-KO neurons may be implicated in spine shrinkage. This may be explained by a previous article showing that SPIN90, a Shank binding partner, regulates dendritic spine morphology (Kim S. M. et al., 2009). Kim S. M. et al. (2009) showed that SPIN90 overexpression causes accumulation of Shank and PSD95 within dendritic spines and mediates spine maturation and spine head enlargements by altering protein composition associated with actin cytoskeleton. Thus, SPIN90 depletion in cells may induce spine shrinkage and decrease in PSD95 puncta density during stimulation such as cLTD. It is interesting to note that spine shrinkage, which is mainly mediated by the LIM Kinase and cofilin cascades is independent mechanism from stimulation-induced AMPAR internalization and LTD (Zhou et al., 2004). Internalization of AMPARs is known to be more specific to phosphatase activity downstream of NMDAR stimulation during LTD. Therefore, we have checked activity (Supplementary Figure S5C) and expression (data not shown) of PP1, the major phosphatase during cLTD, but could not find any differences in them between WT and *Spin90*-KO neurons. Thus, we figured that SPIN90 is involved in a separate pathway modulating LTD, apart from major downstream signaling.

Meanwhile, the increase of synaptic surface GluA2 levels in *Spin90*-KO neurons may be explained by our previous report that *SPIN90* knockdown caused a delay in EGFR endocytosis, whereby the most of the EGFR was detected on the cell surface, even after stimulation (Oh et al., 2013). Overexpression of SPIN90 variants led to the impairments in EGFR-mediated endosome targeting and vesicle formation. Just as *SPIN90* knockdown caused a defect in internalization of EGFR into the cell membrane, and a concomitant increase of EGFR on the surface of the plasma membrane, NMDAR-mediated GluA2 internalization might have been obstructed because of SPIN90 deficiency. Therefore, the difference in basal GluA2 levels between WT and *Spin90*-KO neurons may be attributed to an endocytic pathway specific defect.

To focus on the mechanism underlying AMPAR trafficking within *Spin90*-KO neurons, we then asked what compromised NMDA-induced AMPAR internalization in *Spin90*-KO neurons. PP1 is mainly responsible for dephosphorylation of GluA1 at residue Ser845 (Collingridge et al., 2004). GSK3β, a multifaceted master kinase in many cellular processes, is also regulated by PP1 during LTD (Llorens-Martín et al., 2014). Potent GSK3 inhibitors such as lithium, kenpaullone and SB415286 block PP1-dependent GSK3 activity, which limits GSK3 to facilitate LTD (Peineau et al., 2007). However, in this study, we were unable to detect any interactions of PP1 with SPIN90 in WT neurons, and PP1 activity assays showed no changes in PP1 activity in the absence of SPIN90 (Supplementary Figures S5B,C). Therefore, PP1-GSK3β signaling seemed irrelevant for LTD impairment in *Spin90*-KO neurons. Instead, we did show involvement of the SPIN90-Akt-GSK3β pathway in LTD. GSK3β phosphorylation of PSD95 at Thr19 is believed to disperse PSD95 from synapses, allowing immobilization of AMPARs from PSD95 and LTD (Nelson et al., 2013). However, PSD95 phosphorylation at Thr19 was abolished in cLTD-induced *Spin90*-KO neurons, indicating a defect in PSD95 phosphorylation by GSK3β. Our studies further demonstrated that SPIN90 is able to bind to Akt, and hinders Akt from phosphorylating its downstream effector, GSK3β. Therefore, Akt-GSK3β signaling during LTD may play an essential role in the *Spin90*-KO neuron phenotype (Figure 7). Based on our observations, SPIN90 acts as an auxiliary protein which may sequester Akt activity by interaction (Figure 7). During basal and LTD conditions, our data show that SPIN90 binds to Akt to prevent it from regulating GSK3β activation. GSK3β, in its basal unphosphorylated state, is able to phosphorylate PSD95 (Thr19), which undock AMPARs from PSD95 and allow internalization of AMPARs. In the absence of SPIN90, however, interactions with Akt are lost, which leads to a concomitant sequestration of GSK3β by activated Akt. Collectively, SPIN90 mediates GSK3β activation, thereby coordinating proper AMPAR internalization and LTD.

It is well known that changes in either laminar organization or spine structures result in abnormalities in both synaptic plasticity and behavior (Cremer et al., 1998; Lee, 2014). Interestingly, mutant mice which have abnormal dendritic spines such as Fmr1 KO and Limk1 KO also showed deficits in reversal learning (D’Hooge et al., 1997; Meng et al., 2002; Van Dam et al., 2005). Therefore, we cannot exclude the possibility that the perturbations in cellular architectures of CA1 in *Spin90*-KO mice might be associated with the observed behavioral deficits (Figure 6). The causal link between LTD and behavioral flexibility has been recently established. However, the role of ABPs in the regulation of LTD and behavior has been poorly understood. SPIN90 indirectly regulates GSK3β activity by sequestration of Akt, a key molecular upstream inhibitors of GSK3 during LTD. Ablation of SPIN90 causes an intrinsic activation of Akt-GSK3β signaling, which dysregulates AMPAR endocytosis and concomitant impairment in LTD. Also, SPIN90 appeared to be involved in behavioral modification such that object-place recognition, fear memory extinction, and behavioral flexibility were differentially altered. Thus, these findings indicate a novel role of SPIN90 during plasticity and memory, especially those that are involved in memory processing.

Further exploration will be necessary to determine whether SPIN90 plays a role during LTD regulation and behavioral modification and to find its causal link to Akt signaling. While elimination of SPIN90 impaired long-term recognition memory and cognitive flexibility (Figure 6), whether re-expression of SPIN90 is capable enough to remedy these behavioral deficits is an unresolved question. In addition, it is interesting to note that SPIN90 conjoins the PSD constituent by interacting with Shank1b, Homer1b and PSD95, then maintains its role as an important regulator of actin polymerization until spines mature (Kim S. M. et al., 2009). Importantly, mutations in Shank family proteins (also known as ProSAP) such as Shank1, Shank2 and Shank3 have been implicated in ASD and intellectual disability (Sato et al., 2012; Won et al., 2012; Mei et al., 2016). Interestingly, *Spin90*-KO mice showed comparable sociability in the three-chamber test, an efficient method to examine any reciprocal sociability impairments in rodents with autism-related disorders (Supplementary Figures S3G–I). Nonetheless, it is possible that SPIN90 might have a role in the modulation of autism or ASD, which would be an interesting and important future study. Based on our findings, however, a mechanism for SPIN90 in regulating Akt-mediated LTD is a possible scheme (Figure 7), that is now open for further investigation.

## Author Contributions

DHK, WKS, IHC and C-SP provided substantial contributions to the conceptual design of the study. DHK, YHH, MK, Y-SL and WS drafted the work and prepared the manuscript. DHK and IHC performed biochemical experiments, data acquisition and analysis. DHK and MK handled, bred and genotyped mutant mice and WT mice. C-HK and H-HR performed electrophysiology experiments and analysis. DHK, YHH and KHC performed histological experiments and analysis. DHK and YHH performed and analyzed diolistic labeling experiments. MK performed and analyzed behavioral studies. SR, WKS and Y-SL supervised the work and provided conceptual development. MHK and H-HR cloned and packaged viral vectors. C-SP and SR provided critical advisory input into the drafts and C-SP participated in electrophysiological analysis. All authors above agreed to be accountable for all aspects of the work in ensuring that questions related to the integrity and accuracy of any part of the work are appropriately investigated and resolved. All authors approved of the final version to be published in Frontiers in Molecular Neuroscience.

## Conflict of Interest Statement

The authors declare that the research was conducted in the absence of any commercial or financial relationships that could be construed as a potential conflict of interest.
