# Supplementary material for: SPIN90 Modulates Long-Term Depression and Behavioral Flexibility in the Hippocampus
Source: Front Mol Neurosci. 2017 Sep 20;10:295. doi: 10.3389/fnmol.2017.00295 (PMC5611360; doi:10.3389/fnmol.2017.00295)
Supplement: Supplementary file 1 [file Data_Sheet_1.docx]

Supplementary Material

**SPIN90 modulates long-term depression and behavioral flexibility in the hippocampus**

Dae Hwan Kim^1^, Minkyung Kang^2^, Chong-Hyun Kim^3^, Yun Hyun Huh^1^, In Ha Cho^4^, Hyun-Hee Ryu^2,5^, Kyung Hwun Chung^6^, Chul-Seung Park^7^, Sangmyung Rhee^5^, Yong-Seok Lee^2,*^ and Woo Keun Song^1,*^

^1^Bio Imaging and Cell Logistics Research Center, School of Life Sciences, Gwangju Institute of Science and Technology, Gwangju, South Korea, ^2^Department of Physiology, Department of Biomedical Sciences, Seoul National University College of Medicine, Seoul, South Korea, ^3^Center for Neuroscience, Korea Institute of Science and Technology, Division of Bio-Medical Science and Technology, KIST School, Korea University of Science and Technology, Seoul, South Korea, ^4^Department of Biological Sciences, Dartmouth College, Hanover, USA, ^5^Department of Life Science, Chung-Ang University, Seoul, South Korea, ^6^Electron microscope facility, Dental Research Institute, Seoul National University, Seoul, South Korea, 7School of Life Sciences, Gwangju Institute of Science and Technology, Gwangju, South Korea

**^*^Correspondence to:**

Woo Keun Song, PhD, Bio Imaging and Cell Logistics Research Center, School of Life Sciences, Gwangju Institute of Science and Technology, 123 Cheomdangwagi-ro, Buk-gu, Gwangju, 61005, Republic of Korea, Tel: +82-62-715-2487; Fax: +82-62-715-2543; E-mail: [wksong@gist.ac.kr](mailto:wksong@gist.ac.kr)

Yong-Seok Lee, PhD, Department of Physiology, Seoul National University College of Medicine, 103 Daehak-ro, Jongro-gu, Seoul, 03080, Republic of Korea; Tel: +82-2-740-8225 ; E-mail: [yongseok7@snu.ac.kr](mailto:yongseok7@snu.ac.kr)

## Supplementary Figures

**
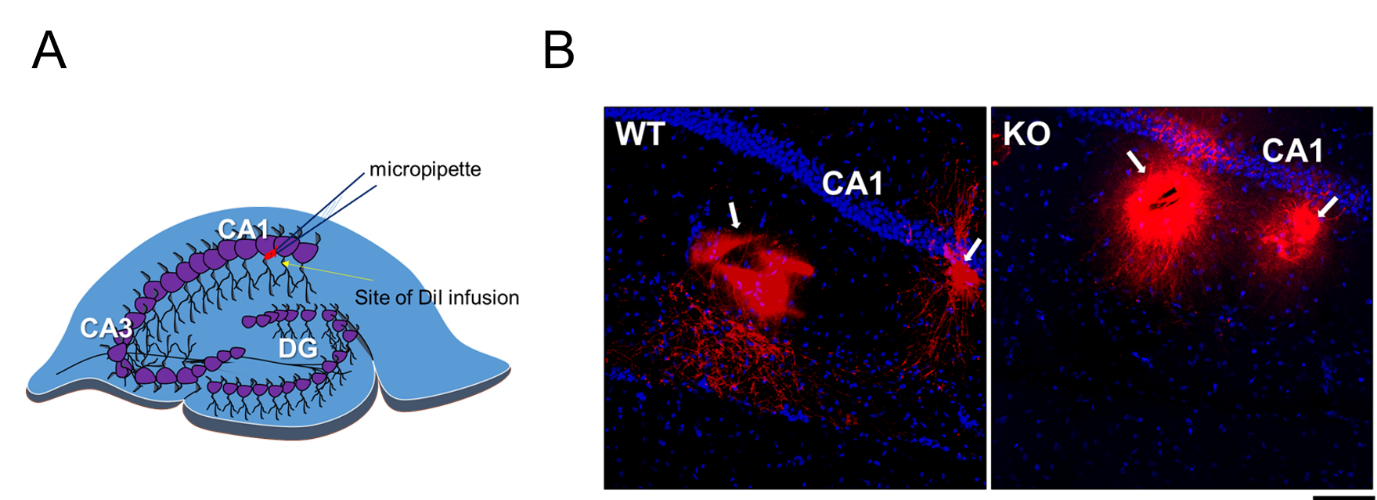
**

**Supplementary Figure S1 | (related to Figure 1)** Diolistic labeling on lightly fixed slices. **(A)** Schematic of extracellular diolistic infusion on lightly fixed hippocampal slices with 1.5% paraformaldehyde (PFA). Tips of micropipettes pulled from borosilicate glasses were coated with 0.2 mg/ml of DiI solubilized in DMSO and applied onto CA1 pyramidal regions. **(B)** Diffusion of dye was allowed for 16 h, after which confocal images of CA1 regions were taken in 20x magnification. DiI-infused neuronal projections were observed in red, and soma were observed in blue, counterstained with DAPI. White arrows indicate sites of infusion. Scale bar indicates 100 μm.

**
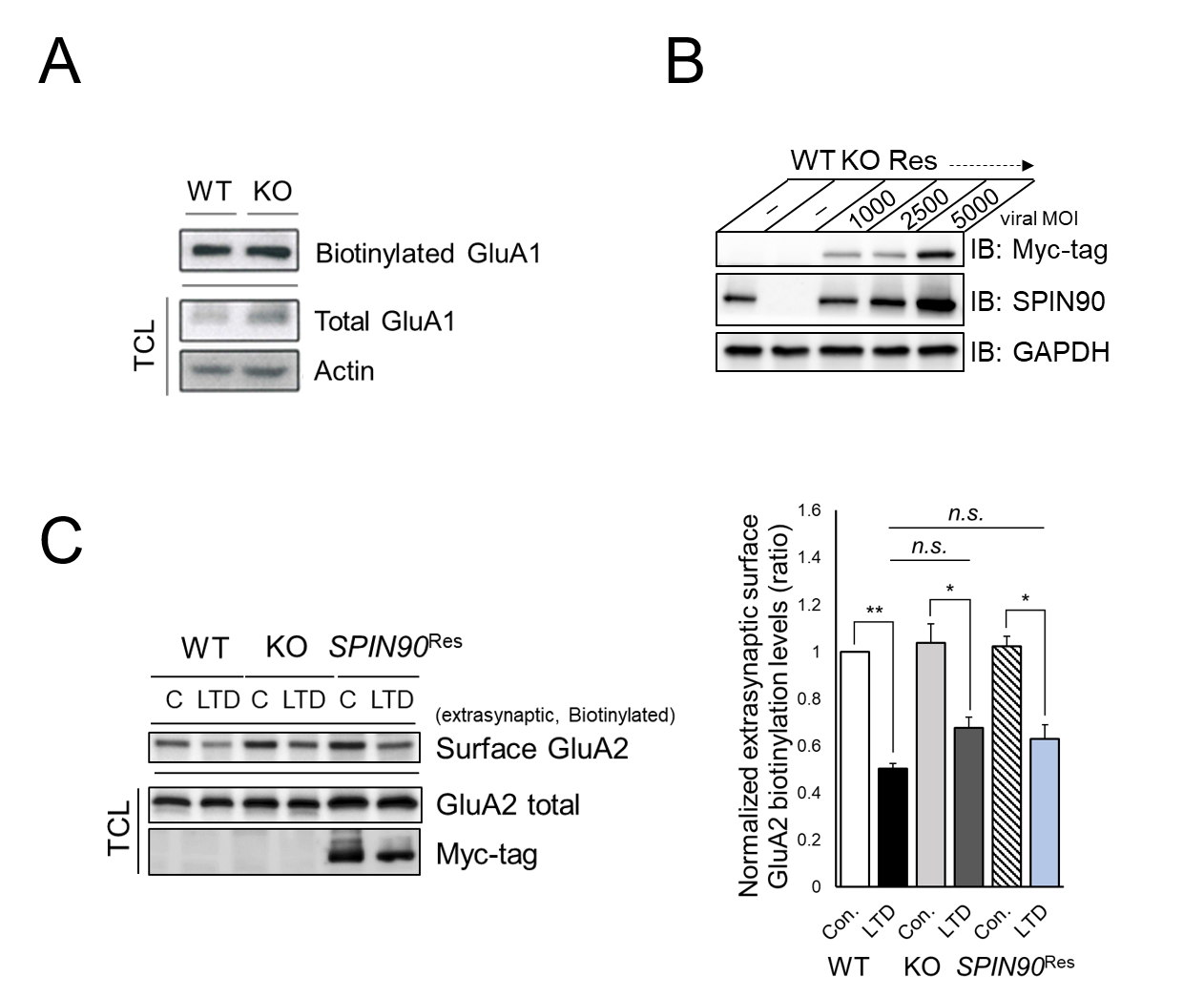
**

**Supplementary Figure S2 | (related to Figure 3)** Composition of AMPAR subunits GluA1, extrasynaptic surface GluA2 and AAV characterization. **(A)** Immunoblot analysis of hippocampal GluA1 composition in WT and *Spin90*-KO neurons. Biotinylated GluA1 levels indicate surface GluA1. **(B)** Determination of viral titers for AAV-6xMyc*-SPIN90*. DIV10-11 cultured hippocampal neurons were infected with AAV-6xMyc*-SPIN90* in a dose dependent manner. The MOI of 1000, 2500, 5000 were used to determine SPIN90 rescue. Myc detecting blots were stripped and reblotted with anti-SPIN90 antibody. **(C)** Biotinylated extrasynaptic surface GluA2 level analysis in WT, *Spin90-KO,* and rescued neurons. TCL indicates total cell lysate. All data are expressed as mean ± SEM (*p < 0.05, **p < 0.01, *n.s.,* non-significant).


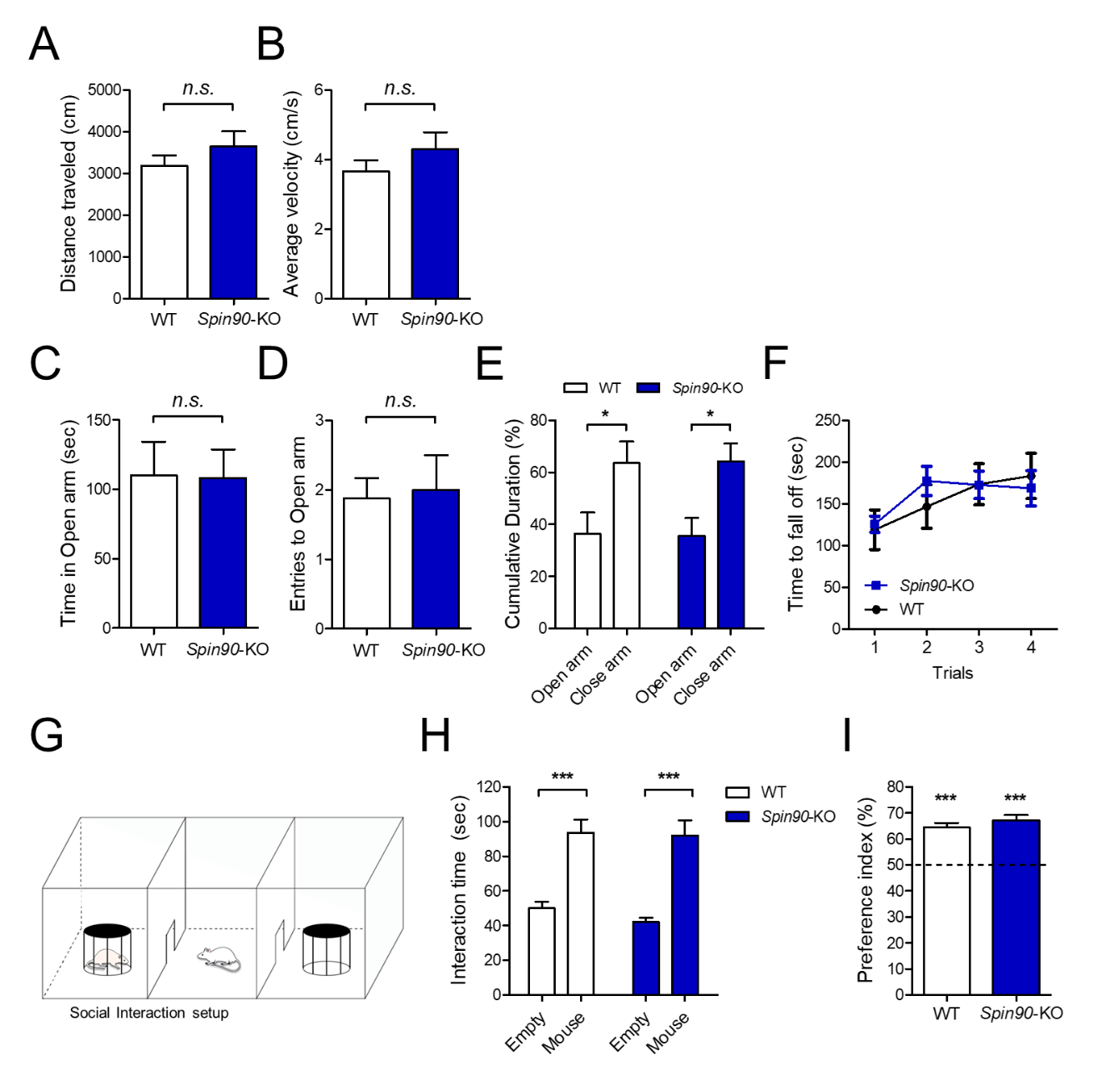


**Supplementary Figure S3 | (related to Figure 6)** Basal locomotion, anxiety and social behaviors of *Spin90-*KO mice. **(A, B)** Overall activity measured in distance and velocity assessed by open field test. **(A)** WT (n = 24) and *Spin90-*KO (n = 27) showed comparable total distance traveled (unpaired t-test, p = 0.3043) and **(B)** average velocity (unpaired t-test, p = 0.3013). *n.s.* indicates non-significant. **(C-E)** Time and entries to open/closed arms assessed by elevated plus maze test. **(C)** WT (n = 8) and *Spin90-*KO (n = 8) showed similar levels of spent time in the open arm (unpaired t-test, p = 0.9587), and **(D)** number of entries to open arm (unpaired t-test, p = 0.8326). **(E)** Both groups spent longer time in the close arm than in the open arm. (unpaired t test, *p = 0.0337; *p = 0.0100) **(F)** Rotarod test measured in time to fall off apparatus. *Spin90-*KO (n = 8) and WT (n = 7) mice comparable in performance. [two-way ANOVA with Bonferroni test, p = 0.8085 (*n.s*.)]. **(G)** Schematics of social interaction setup. **(H, I)** Both WT (n = 16) and *Spin90-*KO (n = 15) mice spent significantly more time to interact with the stranger mouse than the inanimate object (empty cup), confirmed by the preference index (paired t-test, ***p < 0.0001). All data were expressed as means ± SEM.


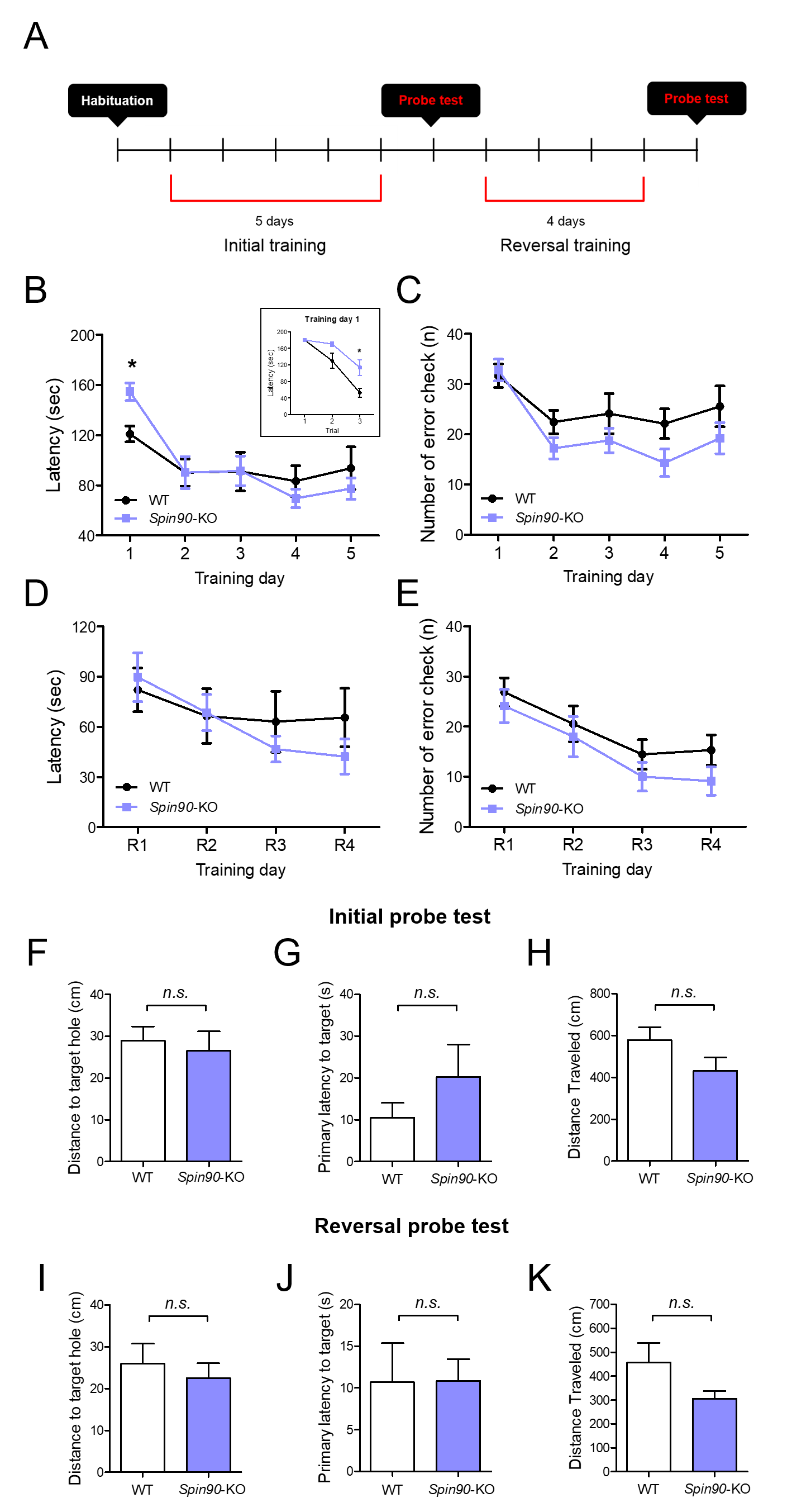


**Supplementary Figure S4 | (related to Figure 6)** Learning in Barnes maze task. **(A)** Experimental design for Barnes maze task. **(B, C)** Latency and number of error checks during training for initial learning. **(B)** WT (n = 9) mice showed significantly shorter latency to escape the maze compared with *Spin90-*KO (n = 9) mice in initial training day 1 (unpaired t-test, *p < 0.01). **(C)** WT and *Spin90-*KO mice showed no significant difference in number of nose poke into errored hole during initial training session. **(D, E)** Latency and number of error checks during training for reversal learning. WT and *Spin90-*KO showed comparable level of **(D)** latency to escape the maze and **(E)** number of error check. **(F-H)** **(F)** Average distance to target hole, **(G)** primary latency to target hole, and **(H)** total distance traveled during the probe test after initial learning were comparable between WT and *Spin90-*KO mice (unpaired t-test, p = 0.6854, 0.2711, 0.1194). **(I-K)** **(I)** Average distance to target hole, **(J)** primary latency to target hole, and **(K)** total distance traveled during the probe test after reversal learning were comparable between WT and *Spin90-*KO mice (unpaired t-test, p = 0.5878, 0.9744, 0.1480). All data were expressed as means ± SEM.


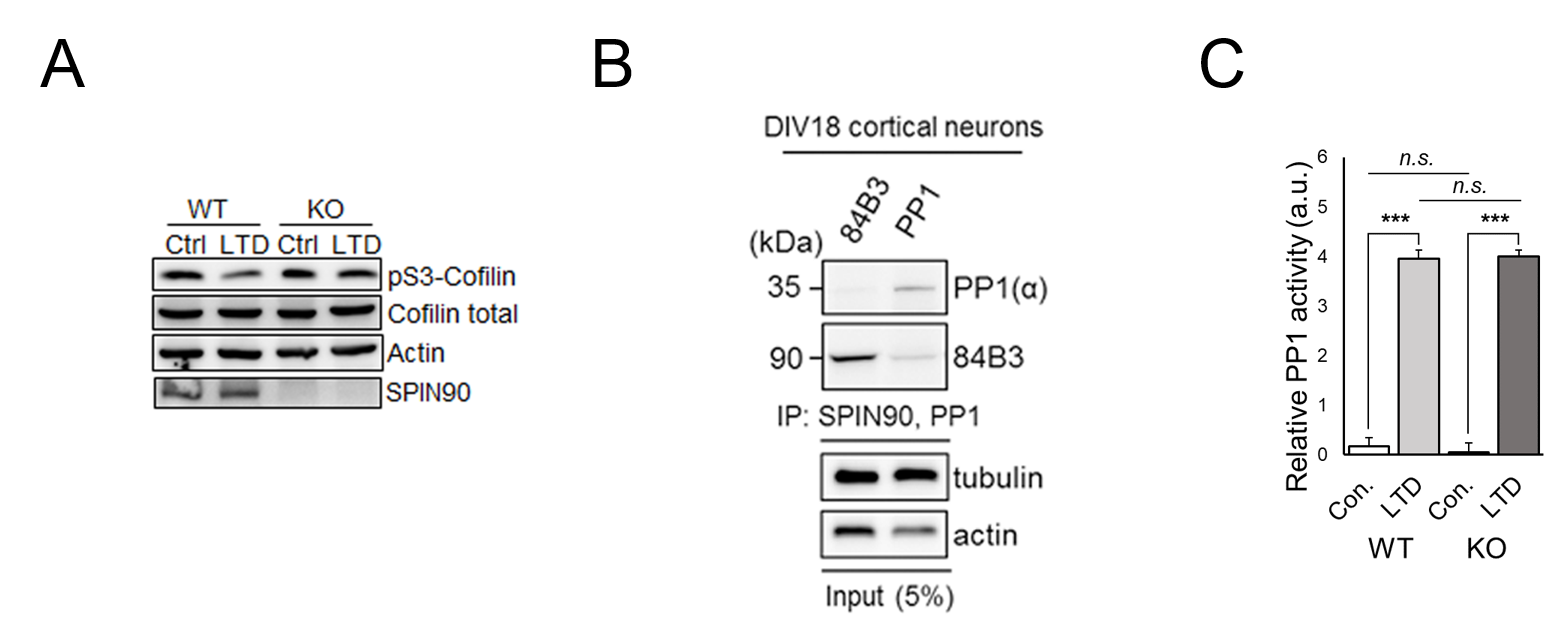


**Supplementary Figure S5 | (described in discussion)** Deficits of LTD in *Spin90-*KO neurons are independent from PP1 regulation. **(A)** Immunoblot analysis of active cofilin (pS3) in hippocampal neurons of both WT and *Spin90*-KO mice. Active cofilin levels were unchanged in *Spin90*-KO neurons after cLTD induction, whereas WT neurons showed normal dephosphorylation and activation of cofilin. **(B)** Immunoprecipitation of PP1 and SPIN90 (84B3) in DIV18 cortical neurons. Weak to no interaction was found between PP1 and SPIN90. **(C)** Relative activity of PP1 in WT and *Spin90*-KO neurons, with or without cLTD induction. PP1 activity levels quantified by ELISA were comparable under basal and post-cLTD conditions for both groups. A.U. indicates arbitrary units. Data were expressed as means ± SEM (n = 3; ***p < 0.001, *n.s.,* non-significant).
